# Heart failure and cancer: From active exposure to passive adaption

**DOI:** 10.3389/fcvm.2022.992011

**Published:** 2022-10-11

**Authors:** Yantao Du, Tao Wu

**Affiliations:** ^1^Ningbo Institute of Medical Science, The Affiliated Hospital of Medical School of Ningbo University, Ningbo, Zhejiang, China; ^2^Department of Cardiovascular Center, The Affiliated Hospital of Medical School of Ningbo University, Ningbo, Zhejiang, China

**Keywords:** heart failure, cancer, active exposure, passive adaption, Cardio-Oncology

## Abstract

The human body seems like a “balance integrator.” On the one hand, the body constantly actively receives various outside stimuli and signals to induce changes. On the other hand, several internal regulations would be initiated to adapt to these changes. In most cases, the body could keep the balance *in vitro* and *in vivo* to reach a healthy body. However, in some cases, the body can only get to a pathological balance. Actively exposed to unhealthy lifestyles and passively adapting to individual primary diseases lead to a similarly inner environment for both heart failure and cancer. To cope with these stimuli, the body must activate the system regulation mechanism and face the mutual interference. This review summarized the association between heart failure and cancer from active exposure to passive adaption. Moreover, we hope to inspire researchers to contemplate these two diseases from the angle of overall body consideration.

## Introduction

The most hazardous and complicated diseases are heart failure and cancer. So far, clinical and basic researchers have formed theoretical principles and related treatments for each disease. However, according to epidemiology studies, heart failure and cancer coexist in a similar population. Although not very comprehensive, the new emerging Cardiac-Oncology has been set up to mainly focus on the heart toxic during the anti-cancer treatment process. Many reviews have given excellent summaries about their associations from the co-incidence, similar risk factors, and correlated regulation mechanisms, which indicated some underlying clues for both diseases (1, 2). Considering each individual as a whole study subject, heart failure and cancer are different manifestations of physical problems but can originate from similar physical backgrounds.

**FIGURE 1:**
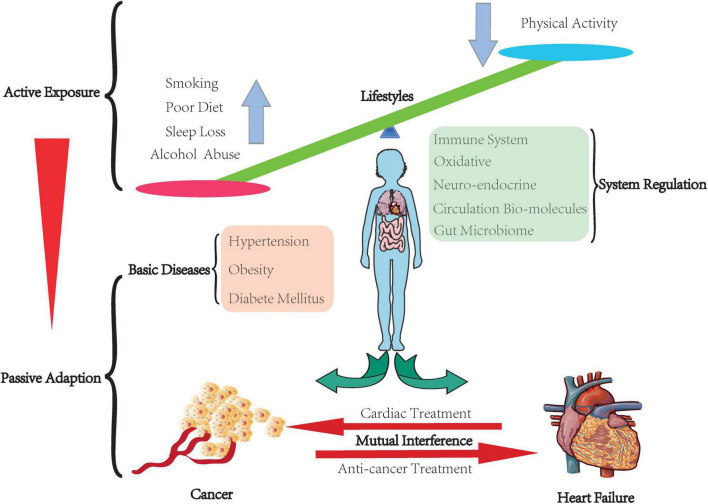
Balance in the human body. The human body keeps the balance through people actively exposing to their lifestyles, which leads to various inter-environment. Some of them have to face their basic diseases. The body has to passively adapt to this inter-environment by arousing the system regulation to correct those temporary unbalance or compensate to a certain pathological state. Furthermore, cancer and heart failure have mutual interference.

Heart failure and cancer populations tend to actively expose themselves to similar lifestyles or have to adapt to their pre-existing physical condition (Figure 1). These stimulations may disturb the primary balance of the individual. They will activate several regulation systems to amend those unbalance, even though some are not physical but pathological, such as the neuroendocrine system, immune system, gut microbiome, and intercellular communication *via* various cytokines and molecules. Furthermore, both could produce mutual interference during their progression and drug treatment. Our review aims to focus on these two diseases and summarize their correlation from active exposure to passive adaptation.

## Active exposure

An unhealthy lifestyle leads to a vast significant influence on the incidence of cardiovascular (CV) diseases and cancer. Unfortunately, many unhealthy lifestyles exist for humans, such as smoking, alcohol addiction, lack of physical exercise, loss of sleep, and poor diet.

Smoking is an essential factor for heart failure and various cancers. On the one hand, tobacco increases the risk of heart failure (HF) by coronary artery disease-dependent mechanisms (3). Continued smoking deteriorates the prognosis of patients with HF, while suspending smoking could decrease the risk of major adverse cardiac events (3). On the other hand, smoking has essential effects on various kinds of cancers, such as lung cancer (4), breast cancer (5), cervical cancer (6), and liver cancer (7). The mechanisms involve several secondary toxicities of tobacco, including irritants, carcinogens, pro-inflammatory stimuli, and oxidizing agents (8). Among them, nicotine contributes to the pathogenesis of CV diseases and cancer (9, 10). It could directly depress apoptosis and promote angiogenesis (8). Likely, smoking-related cancer is also prevalent in patients with CV diseases (11).

Although low-to-moderate alcohol consumption might be beneficial for cardiac function, a chronically large quantity of ethanol consumption is toxic to the heart and vascular, even progressing to heart failure (12). In addition, pre-existing cardiovascular diseases may be deteriorated by alcohol abuse, such as hypertension and cardiomyopathy. Again, about 4% of cancer in the global world is caused by alcohol consumption (13). In addition, it could increase the risk of digestive system cancer and sex-related cancer, such as breast cancer (13). Harriet Rumgay has reviewed the mechanism by which ethanol could produce metabolite to acetaldehyde, which could cause DNA damage and inhibit DNA synthesis and repair (13).

Physical activity has been widely studied as a protection factor for HF patients. Exercise can provide primary prevention for past onset HF and secondary prevention for present HF and can also be used as a prognostic factor for predicting the future of HF patients (14). A meta-analysis result indicated that high levels of total physical activity, leisure-time activity, vigorous activity, occupational activity, walking and bicycling combined, and cardio-respiratory fitness could reduce the risk of heart failure (15). As a comparison, in cancer, a systematic review compared the highest to lowest physical activity levels and summarized that approximately 10–20% risk reduction could be reached in bladder, breast, colon, endometrial, esophageal adenocarcinoma, renal, and gastric cancer (16). Again, according to 18 systematic reviews and meta-analyses, a great deal of physical activity could reduce 40–50% risk of all-cause and cancer-specific mortality in patients with breast, colorectal, or prostate cancer (16). About 40% reduction in cancer incidence and cancer-related death is benefited from increased physical activity (17).

Sleep loss could also result in harmful outcomes, including heart diseases, certain cancers, and all-cause mortality (18–22). According to the data from the health and retirement study in the United States, insomnia symptoms, both cumulatively and individually, are associated with incident HF (23). Sleep loss is also related to various kinds of cancer, such as neck and head cancer (24), prostate cancer (25), and malignant brain tumor (26). Michael et al. reported that sleep loss might activate spontaneous cellular innate immunity (27). They hypothesize that treatments for short sleep duration have the potential to inhibit inflammation and decrease the risk for inflammatory disorders and some cancers in humans (27).

The habit of diet is also crucial for people. Poor diet is present in different ways and contexts. As we all know, some familiar diet habits could cause acute injury and chronic toxicity to the human body. For example, hot food would break down the esophageal mucosa and cause esophageal cancer (28), while moldy food would produce aflatoxin and be associated with liver cancer (29). Furthermore, some new evidence indicated the interaction between diet habits and the human body. For example, an excessive high-fat diet (HFD) would induce toxicity to the heart in rats by promoting cardiac injury biomarker leakage into plasma and altering heart rate and electrocardiogram pattern, as well as plasma ion levels (30). In addition, HFD could induce apoptosis and inflammation in rat hearts, which was supported by detecting higher expression levels of Bax and caspase-3 and a large amount of cardiac cellular DNA fragmentation (30). Similarly, HFD could induce colorectal tumorigenesis by destroying the gut barrier and leading to dysregulation of microbial and metabolomic (31). Besides, excessive intake of red meat is related to cancer and heart failure. According to an umbrella review, red meat consumption was related to a growing risk of overall cancer mortality, including non-Hodgkin lymphoma (NHL), bladder, breast, colorectal, endometrial, esophageal, gastric, lung, and nasopharyngeal cancer (32). A cohort study that involved 29,682 participants found that excessive intake of processed red meat, unprocessed red meat, and poultry, but not fish, was significantly associated with exposure to cardiovascular diseases and all-cause mortality (33). On the contrary, healthy diet patterns could reduce cancer and heart failure incidence, like adequate blood sugar control decreases the incidence of CV disease and cancer (34). Several famous healthy diet patterns could prevent HF. For example, Dietary Approaches to Stop Hypertension (DASH) advocates high potassium and low sodium, sulforaphane (SFA), and total fat (35), while MedDiet (Mediterranean) stresses more unsaturated fatty acids (UFA) (36), which are rich mainly in antioxidants and anti-inflammatory nutrients, and offers a solid and inverse correlation with cardiovascular diseases (37). MedDiet and DASH diets are particularly rich in plant-based foods but limited in processed foods and red meat (38).

## Passive adaption

### Basic diseases: Acceptation of imperfections

Various chronic diseases would force the human body to passively adopt these changes and create a unique background for populations with different diseases.

For the terms of heart, hypertension, obesity, and diabetes mellitus (DM) are harmful to vascular and metabolism, which would finally lead to heart failure (39–47). These changes often start with different interrelated processes but end with HF. Obesity and diabetes mellitus always cause damage to the vascular by inducing inflammation and atherosclerosis to increase vessel stiffness and peripheral vascular resistance because of long-term immersion in high blood fatty acid and sugar. Increased vascular resistance leads to hypertension, forms high pressure for cardiac afterload, and forces myocardial hypertrophy. Furthermore, a high concentration of blood fatty acid and sugar would increase the blood volume, which increases cardiac preload. Overall, the heart always tries to adapt to handle increased preload and afterload, but then it would not endure them, followed by decompensation, leading to heart failure. Some treatments can be used to reverse such dysfunction. For example, strict glycemic control and high-quality insulin therapy could reduce severe cardiac dysfunction in patients with diabetes mellitus (48). Besides, it is interesting that there is a paradox between obesity and HF. Obese people are more likely to develop HF, but they have a survival advantage (49). However, the mechanism is not very clear.

In terms of cancer, although the underlying mechanisms are not very clear, a series of reports supported the significant relationship between chronic diseases and cancer. A large prospective cohort study of over 400,000 subjects indicated that chronic diseases (CV, diabetes, chronic kidney disease, pulmonary disease, and gouty arthritis) were independently related to cancer incidence based on a regular risk score (50). Moreover, the accumulative score of chronic diseases has a dose-dependent relationship with cancer incidence and mortality (50). For example, in patients with hypertension, a 10mmHg increment in blood pressure was associated with an increased risk of cancer incidence (HR 1.07, 95% CI 1.04–1.09) in men and cancer-related mortality (HR 1.12, 95% CI 1.08–1.15 and HR 1.06, 95% CI 1.02–1.11, respectively) both in men and women (51). Obesity is associated with a chronic pro-inflammatory state, which could induce DNA damage and cancer incidence (52). Fatty tissue also plays the role of a sizeable endocrine organ, which could produce a great deal of estrogen and promotes hormone-related cancers, such as ovarian and breast cancer (53). Furthermore, adipose tissue could secrete many adipokines related to cellular survival. For example, leptin was one of the well-known adipokines with cell-proliferative effects (54), while another well-known adipokine, adiponectin, was reported to have anti-proliferative effects (54). Besides, insulin and insulin-like growth factors (IGF-1) were increased in obese subjects. High levels of IGF-1 were reported to relate to the development of cancer (55), which was hypothesized and observed to increase cancer incidence by promoting cell proliferation (56–58). Therefore, lowering weight or reducing the weight loss by surgery would reduce cancer risk (59, 60).

### Systematic adjustment

#### Immune system: Chronic inflammation

The immune system is involved in developing cancer and HF (61, 62). Atsushi Anzai has a well-reviewed immune system in CV diseases (63). There are several stages during the HF process. In the early stage after myocardial infarction (MI), different immune cells move to the injury area and try to constrain and restore the primary damage. While in the late stage, a low grade of chronic activation could induce heart modification (64, 65). First, neutrophils rapidly move to the damaged area and activate a pro-inflammatory phase after MI. The infiltrated neutrophil population changed along with the healing process and gradually acquired surface lectin SiglecF (66). Then, macrophages infiltrate and resolute the necrotic tissue and start the process of scar formation in the coming 3–30 days. In HFpEF (heart failure with preserved ejection fraction) heart, cardiac macrophages induce myocardial cell death and interstitial fibrosis. Stefan Frantz et al. have systematically reviewed the function of macrophages in different stages of ischemic heart diseases (67). Interestingly, macrophages show heterogeneity because of their sources. CCR2^+^ (C-C chemokine receptor ^+^) macrophages come from embryonic, the primary resident population in a healthy heart. However, ischemic cardiac injury induces monocyte-derived CCR2^–^ macrophages to infiltrate the heart (68, 69). Engulfment results in increased fatty acid in macrophages, activate mitochondrial respiration, and initiate anti-inflammatory responses during the wound healing process (68, 70). The following remodeling phase will involve low-grade inflammation and non-infarcted myocardium, which are regulated by cytokines and innate immune receptors. It is a long, complicated process involving not only various kinds of immune cells but also numerous pro-inflammatory cytokines, which are increased and may contribute to the development of HF (71). Activated dendritic cells induce B- and T-cell proliferation by migrating from injury myocardium to pericardial adipose tissue fat-associated lymphoid clusters (FALCs) (66, 68). In response to acute injury of the heart, a group of innate B cells within FALCs expressed a considerable amount of granulocyte-macrophage colony-stimulating factor (GM-CSF), induced interleukin-23 (IL23), and interleukin-17 (IL17) secreted from immune cells (66, 68). However, some regulation mechanisms play protective effects. A subpopulation of macrophages with GATA binding protein 6 (GATA6) expression could inhibit excessive cardiac fibrosis (68), while Group 2 Innate lymphoid cells (ILC2) population stimulated by interleukin-2 (IL2) expanded in pericardial adipose tissue to protect cardiac function (68).

In cancer, the abnormal immune system is also related to cancer progression and metastases (72). Currently, immune therapy focused on the immune checkpoint has obtained spectacular results (73). However, limitations were revealed by the wide use of these drugs. Targeting the tumor immune system may be at the cost of deteriorating the myocardial immune system (74). Meijers et al. reported that new-onset cancer could be predicted by high-sensitivity C-reactive protein and mid-regional pro-adrenomedullin (75).

Inflammation could also play a crossroads between CV diseases and cancer. In Canakinumab Antiinflammatory Thrombosis Outcome Study (CANTOS) trial, the interleukin-1 (IL-1) blocker canakinumab was used to test whether IL-1 inhibition could attenuate coronary events in future (76). Compared with placebo, canakinumab can reduce about 25% of major adverse CV events (HR 0.75, 95% CI 0.66–0.85) (76). More interestingly, treatment with canakinumab could also decrease the incidence of lung cancer and mortality in a significant dose-dependent manner [Incidence: HR 0.33, 95% CI 0.18–0.59, *P* < 0.0001; Mortality: HR 0.23, 95% CI 0.10–0.54, *P* = 0.0002] (77).

#### Oxidative stress

Oxidative stress originates from unbalancing between reactive oxygen species (ROS) generation and antioxidant (78), which was defined as an excessive accumulation of ROS relating to antioxidant defense (78). ROS can be produced through several sources (78). Among them, mitochondria produce ROS by transporting a single electron to molecular oxygen (78). Mitochondrial oxidative phosphorylation provides high energy to support the heart’s function and is involved in cancer progression (79).

In HF, Hill and Singal reported that antioxidant deficits and oxidative stress coexist in patients of HF after MI, which may affect cardiac function (80). Also, ischemia or hypoxia would induce ROS increase and be related to myocyte damage of MI (81). Because of abnormal mitochondrial metabolism in HF, glycolysis increases lactate production in a failing heart (82).

In cancer, altered mitochondrial metabolism promotes the glycolysis to adapt to the rapid proliferate tumor cells. Pyruvate dehydrogenase (PDH) and PDH kinase (PDK) are two key modulators. PDH controls the rate of glucose oxidation, while PDK inhibits PDH (82). Interestingly, PDK upregulation and PDH inhibition are in both HF and tumor cells (83). On the one hand, dichloro (a PDK inhibitor) could enhance PDH activity to lower ischemic injury and improve cardiac function. On the other hand, it could also reduce cancer development (83).

#### Neuroendocrine: Non-coordinated activation

**FIGURE 2:**
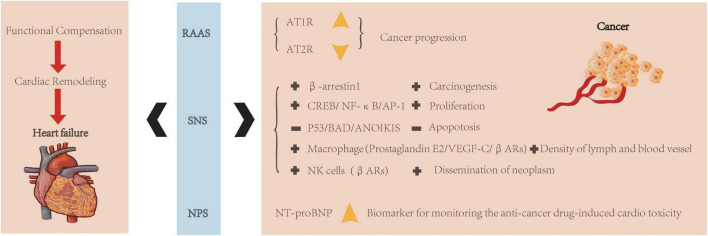
Neuroendocrine regulation of heart failure and cancer. Neuroendocrine system (RAAS, SNS, and NPS) is related to both heart and cancer. They were always aroused to realize functional compensation, but following with cardiac remodeling and then leads to heart failure. In RAAS, increased AT1R and decreased AT2R were associated with cancer progression. In SNS, various kinds of signaling pathways are related to cancer progression (+: increased expression; -: decreased expression). In NPS, NT-proBNP could be a biomarker for monitoring anti-cancer treatment-induced cardiotoxicity.

As we all know, the neuroendocrine system plays the most crucial role in cardiovascular regulation. For the cardiovascular system, there are many neurohormonal pathways, firstly serving as a compensation mechanism, including the renin-angiotensin-aldosterone system (RAAS), sympathetic nervous system (SNS), and natriuretic peptides system (NPS) (Figure 2). They could be promptly activated to react to cardiovascular events when a certain balance was disrupted at a sudden time. Still, gradually chronic neuroendocrine non-coordinated activation contributes to disease progression by promoting cardiac remodeling and deteriorating heart function (84).

However, compared to the heart, local RAAS hormones and receptors differ in various cancers (85). For example, angiotensin II receptor type 1 (AT1R) increased expression during cancer progression, while Ang II/At2R signaling exerts the opposite effect (85) (Figure 2). Over-activity of SNS may result in carcinogenesis *via* the β-arrestin-1 signaling pathway (86). It also could induce cell proliferation through specific molecular pathways, such as cAMP-response element binding protein (CREB), nuclear factor-k-gene binding (NF-kB), and activator protein-1 (AP-1) (87). Besides, it confers resistance to apoptosis through various mechanisms, such as inhibition of p53 (87), proapoptotic protein BAD (bcl2 associated death promoter) (88), and anoikis (89). Because of the broad expression of β1 and β2 ARs (β1 and β2 adrenoceptor agonists) in cancer, β-blockers might be a candidate target for cancer treatment (90, 91). The SNS could also modify the cancer microenvironment (92). For example, in response to the β-adrenoceptor agonist (βAR) stimulation, tumor-associated macrophages release prostaglandin E2 and stimulate vascular endothelial growth factor C (VEGF-C) expression to increase lymph and blood vessel density (93). Moreover, the SNS could suppress natural killer cells by activating βAR activity promoting neoplasms dissemination (94). Besides, the role of natriuretic peptides in carcinogenesis has also been accessed (94). In addition, N-terminal pro-brain natriuretic peptide (NT-proBNP) has been verified to be involved in cancer progression and could play as a biomarker for monitoring anti-cancer drug-induced cardiotoxicity (95).

#### Circulation molecules: Exosome-mediated indirectly regulation

Microvesicles, especially exosomes, play an essential role in various diseases. They bring nuclear acid and protein molecules to participate in cell communication. Exosomal miRNAs are vital in diagnosis biomarkers for certain diseases because of the tissue specificity. For example, in the heart of a patient with heart failure, secreted Exo-microRNA-21-5p damages the regenerative potential of the heart (96). Exo-miR-92b-5p has also been verified as a biomarker for HF (97). In cancer, a massive amount of miRNAs not only regulate cancer progression (98) but also participate in creating the tumor microenvironment. For example, exo-miR-522 derived from cancer-associated fibroblasts inhibited ferroptosis in the cancer cell and led to chemoresistance in GC (gastric cancer) (99). For example, tumor-derived exo-miR-934 can regulate the communication between colorectal cancer cells and tumor-associated macrophages to stimulate colorectal cancer liver metastasis (100).

Because of tissue-specific differences, many kinds of circulation miRNAs were studied as biomarkers of certain diseases, such as heart failure and cancer. However, circulation exosomal miRNAs might affect the whole body. In this review, we summarized the function and mechanism for several kinds of miRNAs and tried to analyze the entire effect of specific miRNAs on both heart and cancer (Table 1).

**TABLE 1:** Whole effects of circulation exosomal miRNAs for both heart failure and cancers.

Exosomal miRNA	Functions in heart failure	Functions in cancers	Several inspired comments or predictions for the whole effects of both hearts and cancer
miR-92b	● miR-92b-5p increased in heart failure as a biomarker (97, 101)	● miR-92b suppressed CD69 on natural killer (NK) cells and predicted the risk of post-transplant HCC (hepatocellular carcinoma) recurrence (102); ● miR-92b was upregulated and could monitor chemoresistance in small lung cancer (103); ● miR-92b decreased in early CRC (colorectal cancer) cancer (104).	● Increased circulation miR-92 in response to chemoresistance might be the toxicity of anti-cancer treatment to the heart.
miR-17-92 cluster (17/18a/20a /19b-1/92a-1)	● miR-19a/19b related with cardiac regeneration (105); ● miR-92a increased in AHF (acute heart failure) (106); ● miR-19b-3p is a biomarker for AH (107).	● miR-17-92 cluster was related to RC (Renal Cell) cancer relapse (108); ● miR-20a-5p was increased and correlated with recurrence of bladder cancer (109); ● miR-17-92 cluster is increased in esophageal adenocarcinoma and is related to progression and lymph node metastasis (110); ● miR-17-5p, diagnosis marker for non-small cell lung cancer (111); ● Tumor-derived Exo-miR-19b-3p promotes M2 macrophage polarization and secrets Exo-LINC00273 to stimulated lung adenocarcinoma metastasis (112); ● miR-19b-3p increased and biomarkers in prostate cancer (113).	● Increased miR-17-92 cluster members were related to the regeneration of heart injury and the promotion of cancer progression. ● Increased miR-19b-3p inducing M2 macrophage polarization might lead to cardiac remodeling in one way and promote cancer progression.
miR-21	● miR-21 could provide a diagnosis of early heart failure (114); ● miR-21 was increased and related to NT-proBNP and galectin-3 levels in acute HF combined with DM (115); ● miR-21 increased in response to an acute exhaustive exercise in CHF (chronic heart failure) patients (116).	● miR-21-5p is associated with angiogenesis and vascular permeability in CR (117); ● miR-21 increased in NSCLC (non-small cellular lung cancer) patients as diagnosis and prognosis (118); ● miR-21 increased in GC patients as a detection marker (119); ● miR-21 elevated in breast cancer as an indicator (120); ● miR-21 upregulated in glioblastoma as a biomarker (121); ● miR-21 unregulated in RCC and decreased after surgery (122); ● miR-21 upregulated in gastric cancer (123).	● miR-21 increased promptly in AHF, and sensitivity to heart function might be associated with its significant role in angiogenesis and vascular permeability, which lead to cancer metastasis and simultaneously induce heart regeneration.
miR-22	● miR-22 increased in heart failure, a biomarker for AHF (106); ● miR-22-5p is higher in HFrEF (heart failure with reserved ejection fraction) patients with AF by altering Ca2 + handling and defective cell-to-cell communication (124).	● miR-22-3p increased in NSCLC and was a biomarker for diagnosis and drug resistance prediction (125); ● miR-22 was a biomarker for osteosarcoma (OS) diagnosis, prognosis, and chemosensitivity prediction (126).	● miR-22 increased circulation could damage the heart function and induce resistance to anti-cancer treatment.
miR-1306	● miR-1306-5p was positively associated with adverse clinical outcomes in AHF (127).	● miR-1306-3p was negatively associated with the TNM (tumor node metastasis) stage of gastric cancer and lymphatic metastasis (128).	● miR-1306 increased indicated bad outcomes for both heart failure and gastric cancer.
miR-30 family (30a/ 30b/30c/30 d/30e)	● miR-30 family members could inhibit Ang II and reduce the expression of inflammation molecules (129); ● miR-30 family decreasing induced increase of CTGF (connective tissue growth factor), which could promote collagen synthesis (130); ● miR-30e-5p was a biomarker for diastolic dysfunction by altering the endothelial cell metabolism and microvascular dysfunction (131).	● miR-30a decreased in osteosarcoma and contributed to chemoresistance (132); ● miR-30 family inhibits breast cancer metastasis (133).	● miR-30 family might be a protective factor for anti–heart failure and cancer because its family members decreased in individuals would lead to heart remodeling and cancer metastasis
miR-106a-363 cluster from X chromosome	● miR-106a was decreased in AHF (134).	● miR-106a was increased in RCC but was decreased after surgery (122); ● miR-106a was increased in breast cancer (135); ● miR-106a was decreased in Cholangiocarcinoma (136); ● Low expression of miR-106a promotes the metastasis of prostate cancer (137).	● The expression level of miR-106 in the heart indicated that it might be involved in cardiac remodeling. Although the reports were inconsistent, according to the newest review of cancer biology (138), the proliferation of cancer cells would be postponed when metastasis started. So miR-106 might be related to tissue repair and proliferation.
miR-1	● Increased in response to carvedilol treatment (139); ● Downregulated in HF, a biomarker for predicting exacerbation of HF (140).	● miR-1 was related to the changes in LVEF (left ventricular ejection fraction) and could be a potential new biomarker of doxorubicin-induced cardiotoxicity in breast cancer patients (141).	● miR-1 was sensitive to heart injury and could be a protective monitor for toxicity of cancer treatment.

#### Gut microbiome: Bidirectional regulation

The human microbiome is composed of various microorganisms (142, 143). Bacteroidetes, Firmicutes, Proteobacteria, and Actinobacteria are the four main bacterial species (144). The human microbial ecosystem is not only composed of a part of the human but also positively participates in human health and disease by regulating the function of the mucosal barrier, immune state, growth of pathological organisms, and metabolisms (145–149).

Accumulating evidence indicated that changes in the gut microbial community were involved in cardiovascular disease (150). *Coriobacteriaceae*, *Erysipelotrichaceae, Ruminococcaceae* (family level), and *Blautia* (genus level) were decreased in chronic HF (151). *Eubacterium rectal and Dorea long catena* from the *Lachnospiraceae* family (152) and *Faecalibacterium* from the *Ruminococcaceae* family were decreasing in older patients (152) and patients with HF (153). In addition, the metabolism derived from the gut is closely related to HF. In 2013, trimethylamine-N-oxide (TMAO) was first reported as a predictor of CV events (154). Decreasing butyrate and increasing TMAO have been consistently verified in heart failure (150). TMAO accumulation stimulates platelet aggregation, promotes foam cell formation, induces inflammation, and reduces reverse cholesterol transport (154–157).

In contrast, the microbiome is also related to the carcinogenesis and progression of various cancers by producing toxic metabolites or carcinogens (158). In addition, it can cause inflammation or immune suppression and indirectly lead to carcinogenesis (158). In patients with CRC, the fecal microbiota belongs to *Bacteroidetes* (mainly *Porphyromonas* and *Prevotella*) and *Firmicutes* (mainly *Enterococcus* and *Streptococcus*) (159). *Helicobacter Pylori* is GC-related bacteria. It can suppress macrophages and T cells *via* protein VacA (159) and inhibit epithelial cell apoptosis (160). Compared to the sterile stomach in healthy individuals, the gut of GC patients harbors a complex microbial ecosystem, including *Proteobacteria, Firmicutes, Actinobacteria, Bacteroides*, and *Fusobacteriaphyla* (161, 162). In liver cancer, a high-fat diet led to the enrichment of Clostridium species and accelerated the progression of liver cancer by producing excess secondary BA deoxycholic acid (163). However, growing evidence showed that bacteria could defend gastroenteric tumors by promoting the host’s anti-tumor immunity (164–167).

**FIGURE 3:**
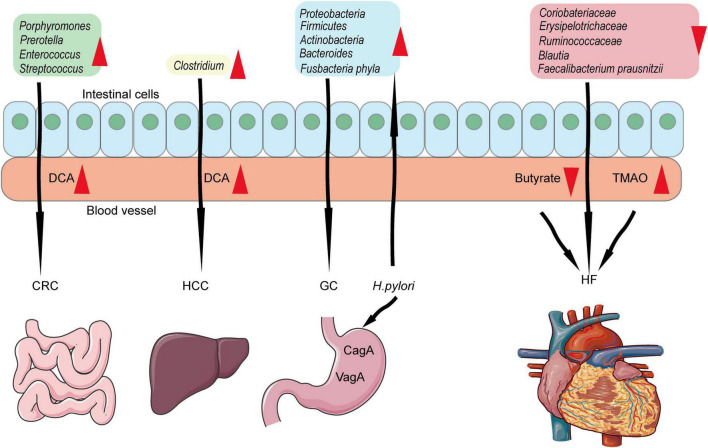
Associations among gut microbiome, cancers, and heart failure. *Porphyromonas, Prevotella, Enterococcus, and Streptococcus* increased in intestinal and produced DCA which lead to CRC formation and progression. *Clostridium* is related to HCC. *Proteobacteria, Firmicutes, Actinobacteria, Bacteroides*, and *Fusobacteriaphyla* are related to GC. Besides, *Helicobacter pylori* could induce GC-related gut microbiome which in turn lead to GC. *Coriobacteriaceae, Erysipelotrichaceae, Ruminococcaceae, Blautia*, and *Faecalibacterium* are related to heart failure by producing TMAO and decreasing butyrate.

Researchers have observed massive data about the gut microbiome changes in certain cancers or heart failure (Figure 3). However, we lack data on cancer combined with heart failure. The gut microbiome could not only indicate the prognosis of cancer and heart failure but also have the potential as probiotics to treat the two diseases. The critical point is that the experimental design cohort should focus on combining both cancers and heart failure so that we can find out the categories and mechanisms of the particular gut microbiome and consciously adjust their component to help deal with both cancers and heart failure.

### Mutual interference

#### The causal relationship between cancer and heart failure

Considering the angle of the individual, it is hard to distinguish the causal relationship between cancer and heart failure. Apart from cancer, infection and multi-organ failure are the second ranks of death in cancer patients (168). However, it is still unclear about the cause of death in advanced cancer (169). Anker et al. have proposed a hypothesis: “advanced cancer is also a heart failure syndrome” in their review (170). They hypothesized that cancer is related to severe tissue inflammation, oxidative stress, and local neurohormonal abnormal activation resulting in heart atrophy, increasing ventricular wall stress, and arrhythmias due to electrical instability and death (170). Furthermore, they hypothesized that heart atrophy might be the tip of the iceberg on the progression of loss of skeletal muscle mass (170). In turn, we can also hypothesize that heart failure is the cause of cancer. Heart failure leads to inadequate organ perfusion, inducing tissue ischemia and hypoxia and promoting carcinogenesis and progression. Some researchers have provided data to support this hypothesis. For example, heart failure patients secrete more circulating factors (serpinA3 and A1, fibronectin, ceruloplasmin, and paraoxonase 1) to promote cancer growth (75). For another, a meta-analysis summarized that heart failure increased the risk of cancer incidence and mortality (171).

#### Toxic to heart during the anti-cancer treatment

In a majority of anti-cancer treatments, no matter whether for common chemotherapeutic drugs or target inhibitors, the effects are not specific to the tumor itself. Their mechanisms are based on cell biology, which processes in every cell, killing cancer and causing organ damage. As we all know, the common side effect is losing hair, vomiting, and losing weight. Besides, the various kinds of toxicity to the heart by anti-cancer treatments are receiving more and more attention in the present study, which leads to the establishment of Cardio-Oncology. In addition, the particular point is that the cardiovascular response to cancer treatment may differ in age and sex (172–174). Large numbers of researchers have reported related heart toxicity mechanisms during anti-cancer treatment. Moreover, many reviews have well summarized this part of the section (175). We tried to list several kinds of drugs and associated mechanisms for heart failure in Table 2.

**TABLE 2:** Cancer therapy-induced heart toxicity.

Anti-cancer therapy	Anti-cancer mechanisms	Side effects on heart
Anthracyclines	● Inhibiting DNA replication and RNA synthesis through inhibition of topoisomerase II (179, 180); ● Damaging DNA, proteins, and cell membrane structure by Chelating iron ions and producing free radicals (181).	● Age and dose-dependent toxicity (182, 183); ● Oxidative stress (181, 184, 185); ● Inhibits fatty acid oxidation (186); ● Interfere cellular energetic buffering and availability of cytoplasmic ATP (187); ● Ferroptosis (188).
Alkylating agent	● Producing highly reactive alkylated groups, interacting with DNA and protein, inhibiting cell proliferation, and inducing cell apoptosis (189, 190).	● Cardiomyocytes energy alteration (191).
ICI (Immune checkpoint inhibitors)	● Inducing anti-cancer response by targeting PD-1/PD-L1, CTLA-4 of T cells (192, 193).	● Myocarditis, pericardial effusion, arrhythmias, acute coronary syndrome, vasculitis (194).
TKI (Tyrosine Kinase Inhibitor)	● Target BCR-ABL (breakpointcluster region-Abelson leukemia virus) tyrosine kinases (195).	● Sex-related: Males are more sensitively to cardiac toxicity than females (172), and females’ hearts showed more fibrosis (173); ● Affect Cxs (Connexins) 43 and 26 and induce cardiomyopathy (172).
PI (Protease Inhibitor)	● Binds selectively and irreversibly to the constitutive proteasome and immunoproteasome (196).	● The mechanisms are not very clear; ● Hypothesis: NF-kB signaling induced apoptosis (196); ● Hypothesis: Down-regulate autophagy and nitric oxide homeostasis (197).
ADC (Antibody Drug Conjugates)	● Combined some cytotoxic drugs with a specific antibody to mediate specific anti-cancer effects (198): HER2 (human epidermal growth factor receptor 2) C-MET (cellular-mesenchymal epithelial transition factor) EGFR (epidermal growth factor receptor) TROP-2 (Trophoblast Cell-Surface Antigen 2) CD30 (TNF receptor superfamily member 8) BCMA (TNF receptor superfamily member 17)	● Inhibition of signal transduction, neoangiogenesis, and repair of DNA damage (199); ● Disrupting ErbB2/ErbB4 (erb-b2 receptor tyrosine kinase 2/erb-b2 receptor tyrosine kinase 4) and NRG-1 (neuregulin 1) signaling pathway (200).
Bispecific antibodies	● Target two or more kinds of sites by cell bridging method (201): ● Anti –VEGF and anti-EGF (anti-epidermal growth factor) (202); anti-DLL4 (anti-delta like canonical Notch ligand 4) and anti-VEGF (203); ● Anti-HER2/CD3 (anti-HER2/CD3 T cell–dependent antibodies) (204); ● Anti-C-MET and anti-PD1 (205); ● Anti-C-MET and anti-PE38KDEL (anti-truncated pseudomonas exotoxin A) (206); ● Anti-4-1BB (anti-TNF receptor superfamily member 9) and anti-PD1 (anti- programmed cell death protein 1) (207); ● Anti-CTLA-4 (anti-Cytotoxic T Lymphocyte-Associated Antigen-4) and anti-PD1 (208); ● Anti-CD29 (anti-integrin subunit beta 1) and anti-CD73 (anti- Ecto-5′-Nucleotidase) (209).	● CRS (Cytokine Release Syndrome) and TLS (Tumor Lysis Syndrome) (210, 211).

Although many side effects on the heart have been found during cancer treatment, some measures can be used to reduce these side effects. For example, empagliflozin (EMPA) could prevent doxorubicin-induced cardiotoxicity (176, 177) by inhibiting ferroptosis, fibrosis, apoptosis, and inflammation through nucleotide-binding oligomerization domain, leucine-rich repeat and pyrin domain-containing 3 (NLRP3) and myeloid differentiation factor 88 (MyD88)-related pathways (178). Such combined treatment is deserved to be encouraged to make more attempts.

#### Cancer risk and prognosis during the cardiac-related treatment

It is challenging to clarify the relationship between CV drugs and cancer risk. According to a large meta-analysis, angiotensin receptor blockers (ARBs), angiotensin-converting enzyme inhibitors (ACEIs), β-blockers, diuretics, and calcium channel blockers (CCBs) caused 5–10% cancer risk or cancer-related death (212). However, in the other two meta-analyses, data showed an uncertain result. In type 2 DM, the overall occurrence of cancer was negatively related to losartan [odds ratio (OR) 0.78, 95% CI 0.63–0.97], but was positively related to candesartan (OR 1.79, 95% CI 1.05–3.06) and telmisartan (OR 1.54, 95% CI 0.97–2.43) (213). Although aspirin was reported to prevent adverse events in CV diseases (214), low-dose aspirin in a low –medium CV risk population was not associated with a lower cancer incidence (215). The mechanisms of aspirin are different in CV diseases and cancer. Antiplatelet effects are the prominent mechanism of aspirin in CV diseases, while cyclooxygenase-dependent and independent mechanisms play the leading roles in cancer (216). Compared with control, low-dose aspirin was related to markedly higher sensitivity for detecting advanced colorectal neoplasms (217). In this review, we listed several main cardiovascular drugs and cancer risk mechanisms in Table 3.

**TABLE 3:** Cardiovascular disease treatment and cancer risk.

Research	Observation region	Cohort design	Treatments	Cancer type	Cancer risk/Prognosis /Mechanisms
Jinhui Li (218)	Hong Kong	6592 anti-hypertensives users and 84,116 anti-hypertensives users with aspirin	Anti-hypertensives (ACEi/ARB, CCB, β-blocker,α-blocker) and (or) aspirin	Lung cancer	Reducing the risk of lung cancer during the anti-hypertensives exposure period.
Seung-Hwa Lee (219)	Korea	207,794 patients	ACEI, ARB	Lung cancer	No difference in lung cancer incidence between patients treatment with ACEI and patients treatment with ARB
Diana R Engineer (220)	Houston	262/454 patients	ACEI,ARB,BBs	Stage III to IV CRC	Exposure to a combination of ACEI/ARB + BB is related to increased survival, decreased hospitalizations, and reduced tumor progression in advanced colorectal cancer.
Shih-Yi Lin (221)	Taiwan	22384 patient	ACEI,ARB	lung cancer	Compared with ARB, ACEI increased the risk of lung cancer. Compared with non-ARB users, ARB users decreased the risk of lung cancer.
Phyo T Htoo (222)	US	532 CRC/111,533 ACEI/ARB initiators	ACEI/ARB initiators	CRC	No association between ACEI/ARB initiation and the short-term CRC risk.
Oliver William Scott (223)	New Zealand (NZ)	14,976 women	β-blocker	Breast cancer	BB-associated risk happened in the initial few months. Long-term BB use may be associated with a lower incidence of breast cancer.
Lina Jansen (224)	Germany	1762 CRCs vs. 1708 controls	β-blocker	CRC	Beta-blocker use is not related to decreased risk of CRC. Long-term beta-blocker use and the risk of stage IV CRC have a positive relationship.
Lovisa Ekestubbe (225)	Sweden	9254 patients	Metoprolol, atenolol, bisoprolol, and other beta-blockers.	CRC	No statistically significant difference in the risk of 90-day postoperative mortality between common β-blockers.

## Summary

This review discussed the relationships between heart failure and cancer from active exposure to passive adaption. People actively select various lifestyles, such as smoking habits, anchor, diet, sleep, and physical activity. These would construct a specific internal environment to passively adapt to these stimuli and try to keep the balance of each individual by inducing various regulation systems, like the neuroendocrine system, immune system, gut microbiome, and intercellular molecules communication through microvesicle transportation. Besides, clinical treatments used in heart failure or cancer could also cause mutual interference with each other. The above series of outer exposures and inner system responses will help us better understand why these two complicated diseases always exist in a similar population and how to coordinate various treatments for both diseases. The best aim for curing heart failure or cancer is not only to reduce the side effect to the lowest level but also to reach a win–win situation for both heart failure and cancer.

## Author contributions

YD contributed to the concept of review and wrote the manuscript. TW contributed to the concept of review and revised the manuscript. Both authors contributed to the article and approved the submitted version.
